# Supplementary material for: Photodegradation of Methylene Blue and Rhodamine B Using Laser-Synthesized ZnO Nanoparticles
Source: Materials (Basel). 2020 Sep 30;13(19):4357. doi: 10.3390/ma13194357 (PMC7579469; doi:10.3390/ma13194357)
Supplement: Supplementary file 1 [file materials-13-04357-s001.pdf]

materials

Supplementary Information

Photodegradation of Methylene Blue and Rhodamine B Using Laser-Synthesized ZnO Nanoparticles

Damjan Blažeka [1], Julio Car [1], Nikola Klobučar [1], Andrea Jurov [2,3], Janez Zavašnik [2], Andrea Jagodar [4], Eva Kovačević [4] and Nikša Krstulović [1,*]

1 Institute of Physics, Bijenička cesta 46, 10000 Zagreb, Croatia; dblazeka@ifs.hr (D.B); jcar@ifs.hr (J.C); nklobucbbb@gmail.com (N.K)
2 Department of Gaseous Electronics, Jožef Stefan Institute, Jamova cesta 39, SI-1000 Ljubljana, Slovenia; andrea.jurov@ijs.si (A.J.); janez.zavasnik@ijs.si (J.Z.)
3 Jožef Stefan International Postgraduate School, Jamova cesta 39, SI-1000 Ljubljana, Slovenia
4 GREMI, UMR7344 CNRS/Université d'Orléans, F-45067 Orléans, France; andrea.jagodar@univ-orleans.fr (A.J.); eva.kovacevic@univ-orleans.fr (E.K.)
* Correspondence: niksak@ifs.hr; Tel.: +385 1 4698 803

Received: 4 September 2020; Accepted: 28 September 2020; Published: date

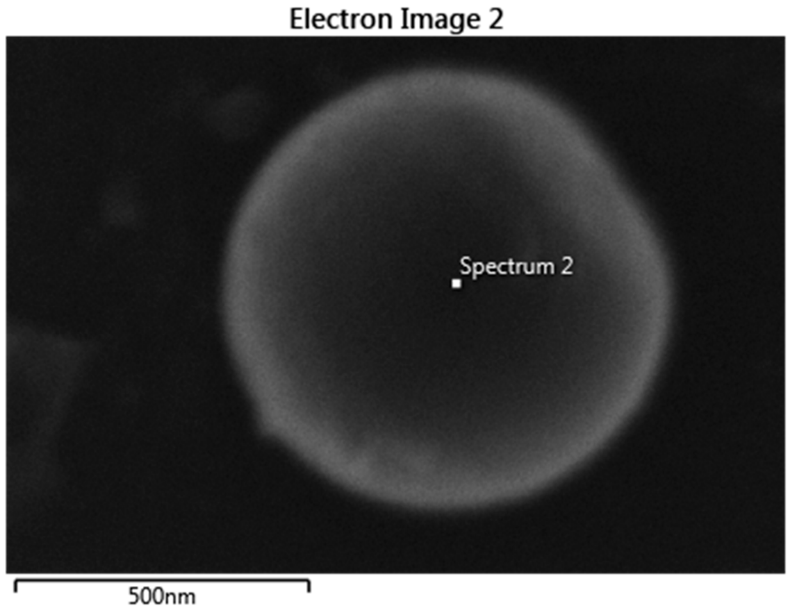

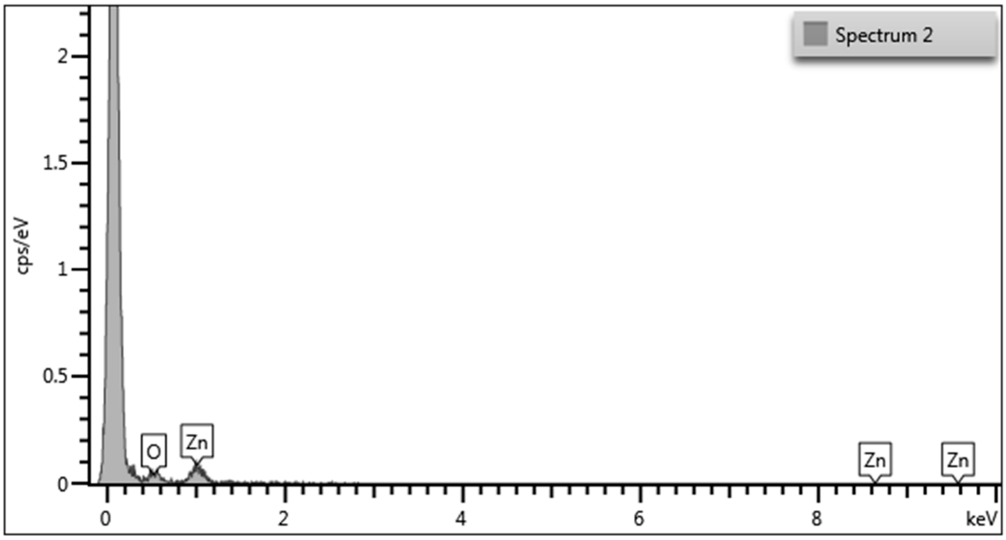

Element	Atomic %
O	47.89
Zn	52.11
Total	100.00

Figure S1. EDS measurements of ZnONP.

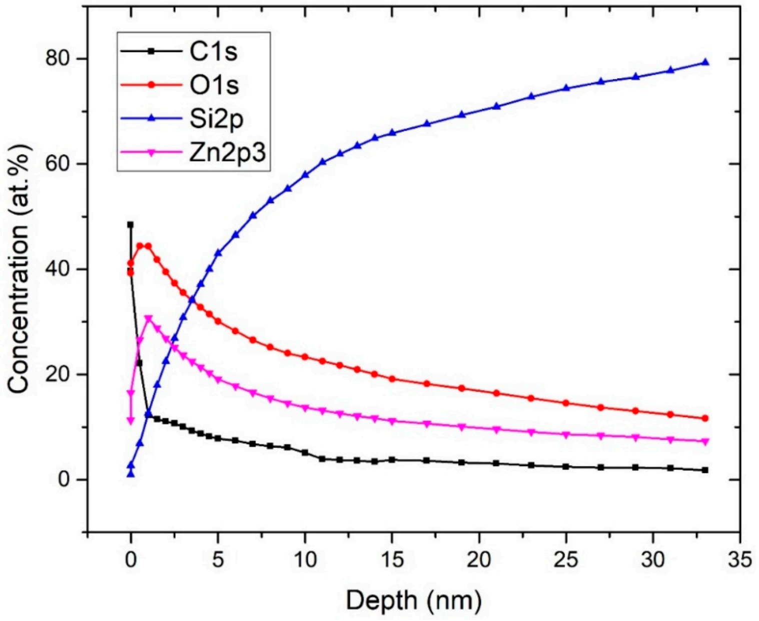

Figure S2. XPS depth profile of the layer of ZnO on Si substrate.

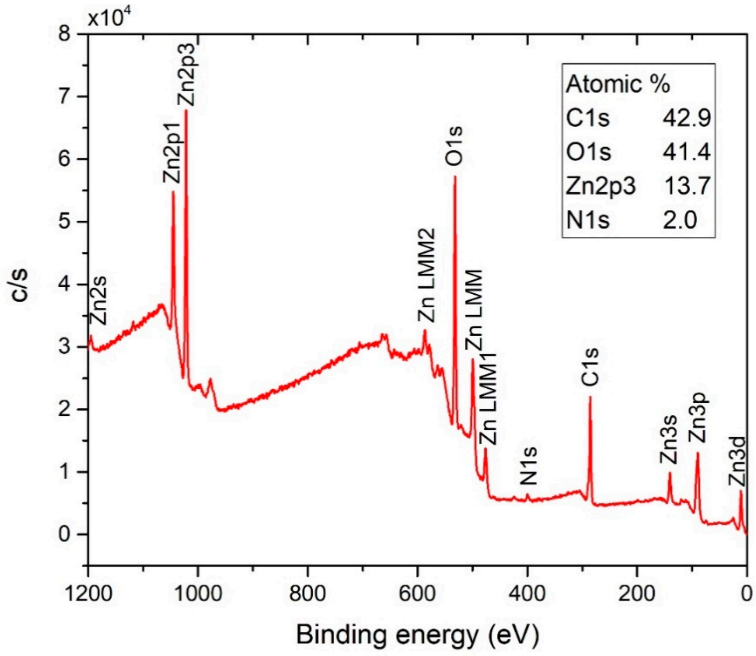

Figure S3. XPS survey spectrum of ZnO.

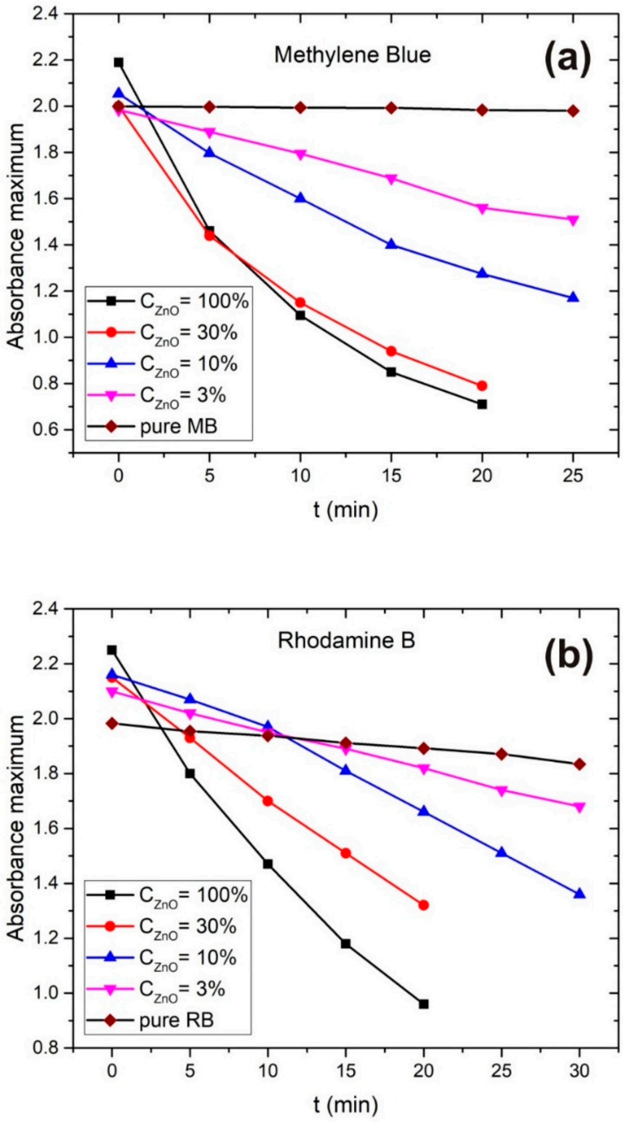

Figure S4. Photoabsorbance peak dependence on irradiation time for different ZnO mass concentrations of (**a**) Methylene Blue and (**b**) Rhodamine B.

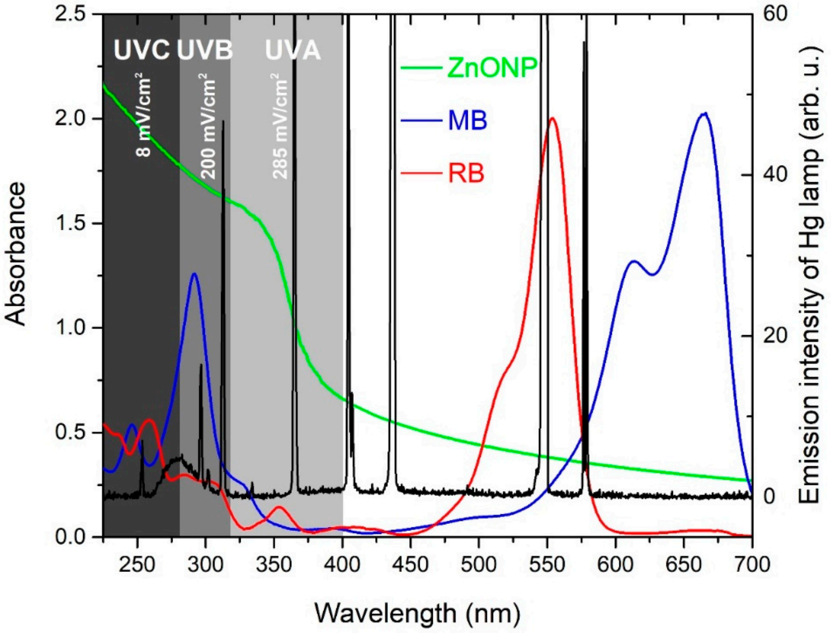

Figure S5. Absorbance spectra of pure ZnONP colloid, MB and RB in comparison with Hg lamp emission spectrum.

© 2020 by the authors. Licensee MDPI, Basel, Switzerland. This article is an open access article distributed under the terms and conditions of the Creative Commons Attribution (CC BY) license (http://creativecommons.org/licenses/by/4.0/).
